# Rarity of Influenza A Virus in Spring Shorebirds, Southern Alaska

**DOI:** 10.3201/eid1408.080083

**Published:** 2008-08

**Authors:** Kevin Winker, Erica Spackman, David E. Swayne

**Affiliations:** * University of Alaska Museum, Fairbanks, Alaska, USA; † US Department of Agriculture, Athens, Georgia, USA

**Keywords:** disease ecology, wild birds, migration, letter

**To the Editor:** Knowledge of avian influenza (AI) virus and its host epidemiology and ecology is essential for effective monitoring and mitigation (*1*). Applicability of global and continental-scale models will be key for expanding this knowledge base. Research in the Delaware Bay area, eastern United States, suggests an ecologic and epidemiologic viewpoint of AI virus in wild birds in which shorebirds (family Scolopacidae) are predominant hosts in spring; however, research in Alberta, Canada, suggests that waterfowl are such in autumn (*2*,*3*). AI virus surveillance in Europe (*4*) suggests that the spring aspect of this scenario does not apply there. To increase knowledge of AI transport among shorebirds in spring in the North Pacific, we conducted AI virus surveillance during the springs of 2006 and 2007 at the Copper River Delta area of Alaska. Millions of birds congregate at this location in the spring, resulting in the highest spring shorebird concentrations in the New World (*5*). We also sampled gulls (Laridae), which are common and heretofore unsurveyed for AI in this ecosystem.

In 2006 and 2007, 1,050 shorebirds (Western Sandpiper, *Calidris mauri*, and Least Sandpiper, *C. minutilla*) and 770 Glaucous-winged Gulls (*Larus glaucescens*) were sampled during peak spring migration at Hartney Bay, Cordova, Alaska (60°28′N 146°8′W; Table). Fresh fecal samples were obtained from tidal flats within <1 to 90 min after identified flocks were dispersed, and samples were placed in sterile medium (brain heart infusion buffer with 10,000 U/mL penicillin G, 1 mg/mL gentamicin, and 20 μg/mL amphotericin B) and either kept cool (<1 week) before transport to Fairbanks (2006) or placed into liquid nitrogen within 2 h of collection (2007). Samples were stored at –70° C; shipped frozen overnight to Athens, Georgia; and maintained frozen until analyzed.

Samples were screened by real-time reverse transcriptase–PCR (RT-PCR) for influenza A virus, and virus isolation was performed on samples that were positive. RNA was extracted by adding 250 μL of sample to 750 μL Trizol LS reagent (Invitrogen, Inc., Carlsbad, CA, USA). Samples were mixed and incubated at room temperature for 10 min. A total of 200 μL of chloroform was then added, incubation was continued for 5 min, and samples were centrifuged for 15 min at 12,000 × *g* at 4° C. Supernatant was removed, and 50 μL was extracted with the MagMax AI/ND viral RNA extraction kit (Ambion, Inc. Austin, TX, USA). RNA was tested for AI virus matrix (M) gene. A positive test result for this gene indicates the presence of any influenza viruses (*6*) when an internal positive control is used (*7*). Positive samples were processed for virus isolation in embryonated chicken eggs by standard methods (*8*). Real-time RT-PCR results were corroborated by processing 50 randomly selected negative samples for virus isolation with 3 egg passages.

Screening for AI virus was conducted on 1,820 samples (Table). Among these, 1 AI virus was identified (A/Glaucous-wingedGull/AK/4906A/2006; H16N?), reflecting an overall prevalence of 0.055% (0% in shorebirds and 0.13% in gulls).

**Table:** Species and sample sizes of wild bird hosts screened for avian influenza virus, Cordova, Alaska, May 2006 and May 2007

Species	Sample size		
	2006	2007	Total
Western sandpiper (*Calidris mauri*)	500	300	800
Least sandpiper (*C. minutilla*)	0	250	250
Glaucous-winged gull (*Larus glaucescens*)	100	670	770
Totals	600	1,220	1,820

Results of power analysis (*9*) suggested that our shorebird samples would detect infection rates >0.9% with 99% probability (95% probability of detecting rates 1%–2% or higher in each year). In gulls, probability of detecting infection rates >1% across both years of the study (>6% in 2006 and >1%–2% in 2007) was 95%.

Virus prevalence in spring shorebirds in Alaska was substantially lower than prevalence in spring shorebirds in the Delaware Bay area (*3*) and more similar to prevalence in spring shorebirds in Europe (*4*). Our shorebird samples (1,050) were fewer than those in other studies (*3;* 4,266 samples from 4 species over 16 years, and *4;* 3,159 samples from 47 species over 8 years, with 35% from spring), representing 25% and 33% of those studies, respectively. Our study covered only 2 years, but it would detect AI virus infections in shorebirds at rates >1%–2% within each year with 95% probability and at rates >0.9% across years with 99% probability. Thus, the prevalence rate among Copper River Delta shorebirds in our study is lower than that found in the 16-year Delaware Bay study (*3*). In the Delaware Bay area, 4 shorebird species were sampled: 3 *Calidris* and 1 *Arenaria* (*3*). Precise statistics are unavailable, but the average 16-year prevalence rate was 14.2%, fluctuating annually from ≈2% to ≈38% (*3*).

In Europe AI viruses were absent among spring shorebirds (*4*). Differences in prevalence rates found among studies may be influenced by species sampled, sampling procedures, and seasonal timing (*4*). However, with >1,000 spring shorebirds sampled, results suggest that differences might exist between the world’s major migration systems (*3*,*4*).

Our results corroborate other recent results (*10*) suggesting that AI prevalence rates among shorebirds at Delaware Bay are not typical within North America. Present evidence indicates (this study; *3,10*) that the role of shorebirds in AI virus ecology and epidemiology is heterogeneous within North America and within a genus (*Calidris*). These findings confirm that knowledge of how AI viruses cycle in wild bird hosts remains incomplete at continental and family-level taxonomic scales. Only further surveillance can fill these knowledge gaps.

## References

[1] Olsen B, Munster VJ, Wallensten A, Waldenström J, Osterhaus AD, Fouchier RA. Global patterns of influenza A virus in wild birds. Science. 2006;312:384–8. 10.1126/science.1122438 16627734

[2] Kawaoka Y, Chambers TM, Sladen WL, Webster RG. Is the gene pool of influenza viruses in shorebirds and gulls different from that in wild ducks? Virology. 1988;163:247–50. 10.1016/0042-6822(88)90260-7 3348002

[3] Krauss S, Walker D, Pryor SP, Niles L, Chengchong L, Hinshaw VS, Influenza A viruses of migrating wild aquatic birds in North America. Vector Borne Zoonotic Dis. 2004;4:177–89. 10.1089/vbz.2004.4.177 15631061

[4] Munster VJ, Baas C, Lexmond P, Waldenström J, Wallensten A, Fransson T, Spatial, temporal, and species variation in prevalence of influenza A viruses in wild migratory birds. PLoS Pathog. 2007;3:e61. 10.1371/journal.ppat.0030061 17500589 PMC1876497

[5] Bishop MA, Meyers PM, McNelley PF. A method to estimate migrant shorebird numbers on the Copper River Delta, Alaska. J Field Ornithol. 2000;71:627–37.

[6] Spackman E, Senne DA, Myers TJ, Bulaga LL, Garber LP, Perdue ML, Development of a real-time reverse transcriptase PCR assay for type A influenza virus and the avian H5 and H7 hemagglutinin subtypes. J Clin Microbiol. 2002;40:3256–60. 10.1128/JCM.40.9.3256-3260.2002 12202562 PMC130722

[7] Das A, Spackman E, Senne D, Pedersen J, Suarez DL. Development of an internal positive control for rapid diagnosis of avian influenza virus infections by real-time reverse transcription–PCR with lyophilized reagents. J Clin Microbiol. 2006;44:3065–77. 10.1128/JCM.00639-06 16954228 PMC1594697

[8] Swayne DE, Senne DA, Beard CW. Avian influenza. In: Swayne DE, Glisson JR, Jackwood MW, Pearson JE, Reed WM, editors. A laboratory manual for the isolation and identification of avian pathogens. 4th ed. Kennett Square (PA): American Association of Avian Pathologists; 1998. p.150–5.

[9] Gregorius H-R. The probability of losing an allele when diploid genotypes are sampled. Biometrics. 1980;36:643–52. 10.2307/2556116 7248433

[10] Hanson BA, Luttrell MP, Goekjian VH, Niles I, Swayne DE, Senne DA, Is the occurrence of avian influenza virus in Charadriiformes species and location dependent? J Wildl Dis. 2008;44:351–61. 18436667 10.7589/0090-3558-44.2.351

